# High-accuracy model recognition method of mobile device based on weighted feature similarity

**DOI:** 10.1038/s41598-022-26518-y

**Published:** 2022-12-18

**Authors:** Ruixiang Li, Xiuting Wang, Xiangyang Luo

**Affiliations:** 1 State Key Laboratory of Mathematical Engineering and Advanced Computing, Zhengzhou, China; 2 Key Laboratory of Cyberspace Situation Awareness of Henan Province, Zhengzhou, China; 3 Henan Polytechnic Institute, Nanyang, China

**Keywords:** Computer science, Information technology

## Abstract

Accurately model recognition of mobile device is of great significance for identifying copycat device and protecting intellectual property rights. Although existing methods have realized high-accuracy recognition about device’s category and brand, the accuracy of model recognition still needs to be improved. For that, we propose Recognizer, a high-accuracy model recognition method of mobile device based on weighted feature similarity. We extract 20 features from the network traffic and physical attributes of device, and design feature similarity metric rules, and calculate inter-device similarity further. In addition, we propose feature importance evaluation strategies to assess the role of feature in recognition and determine the weight of each feature. Finally, based on all or part of 20 features, the similarity between the target device and known devices is calculated to recognize the brand and model of target device. Based on 587 models of mobile devices of 17 widely used brands such as Apple and Samsung, we carry out device recognition experiments. The results show that Recognizer can identify the device’s brand and model than existing methods more effectively. In average, the model recognition accuracy of Recognizer is 99.08% (+ 9.25%↑) when using 20 features and 92.08% (+ 29.26%↑) when using 13 features.

## Introduction

Mobile devices bring great convenience to users’ life. For instance, users can use their mobile device to perform online payment, watch videos, and even use the mobile device to control the home air conditioner remotely^1^. The convenience characteristic is also an important factor for the rapid growth of mobile devices. Currently, there are many legal mobile device manufacturers, such as Apple, Samsung, Xiaomi, etc., and some illegal manufacturers who produce copycat devices for profit. These illegal copycat devices harm consumers’ interests, bring network security risks^2^, disrupt fair competition in the market, and pose a great challenge to the intellectual property protection.

Device recognition is an important technology to obtain device’s category, brand, model, service, version or other information^3^. This technology is significant for cyber asset inventory and security risk assessment^4–6^. The methods recognizing the special model of mobile device accurately can be used in identifying copycat devices, determining the illegal facts of illegal manufacturers^7^ and protecting the intellectual property rights of legal device manufacturers.

The existing device recognition methods are mainly based on the differences between different devices in network traffic, device information in Internet resources, or the physical attributes. Based on those differences, existing methods recognized the type of device by constructing fingerprint database or trained classification model. According to the source of device information, the existing recognition methods can be divided into traffic-based device recognition methods, network-search-based device recognition methods, and physical-attributes-based device recognition methods. We will describe those methods in detail in “Related work” section.

Among the three sources, traffic is the easiest to obtain. However, because the same kind of devices in the same manufacturers use same network protocol for data transmission commonly, the accuracy of model recognition of IoT device using traffic is limited. Physical attributes of different device are different often, which makes it possible to effectively recognize the model of device based on physical attributes, but acquiring physical attributes of devices on a large scale is difficult.

In this paper, we construct a feature set of 20 features, which are extracted from traffic (traffic features include GPU model, resolution, operating system and others) and physical attributes (such as device size and screen size as features). Recognizer identifies the model of target mobile device based on the features in the feature set. When some features of target device may not be available in the realistic work, Recognizer can still effectively recognize the model of mobile devices based on part features in the feature set.

Although we use some physical attributes in Recognizer, there are some applied scenarios in safeguarding the rights of consumers and the intellectual property of legitimate device manufacturers. For examples, for consumers, after purchasing a mobile phone, they are able to obtain the physical attributes and traffic features of the mobile phone, and identify whether the mobile phone is copycat device using Recognizer; regulators can use Recognizer to identify the mobile phones being sold at enforcement sites, thereby determining and collecting evidence of the violations, and protecting the intellectual property of legal device manufacturers.

The main contributions in this paper are as follows:

1. *We build a recognition feature set* Not only Recognizer, but also existing methods can use those features in feature set to recognize the model of mobile device.
2. *We design new device feature similarity calculation rules* According to the form of expression, the features are divided into two types: numerical features and string features. The similarity calculation rules are designed for the two types of features, which realizes the rapid measurement of the similarity between devices.
3. *We propose device feature importance evaluation strategies* We propose RFBR and RFMR strategies to assess the role of feature in recognition, as to select recognition features and determine the feature’s weights. Compared with existing strategy in which all feature weights are same in device recognition, it is more reasonable to determine weight according to the feature importance.
4. *We propose Recognizer to recognize the model of mobile device* Recognizer is able to use features in the feature set to recognize the model of target mobile device. When using all 20 features in feature set, the average recognition accuracy of Recognizer is 99.08%, an improvement of 9.25% over existing methods. And when using any 13 features in the feature set, the average recognition accuracy of Recognizer is 92.08%, an improvement of 29.26% over existing methods. We believe that Recognizer has good application prospects.

The remainder of this paper is organized as follows: In “Related work” section, we will introduce the existing IoT device recognition methods. In “Our method: Recognizer” section, the principle and steps of Recognizer will be introduced in detail. The validity of RFBR and RFMR will be analyzed in “Analysis of Recognizer” section. In “Results and analysis of experiments” section, we will performance experiments to verify the effectiveness of Recognizer. In Recognizer, we use some physical features, but there may be a limit to obtain all physical features at the same time in actual. Therefore, we will discuss the relationship between recognition accuracy and the number of features in “Results and analysis of experiments” section. Finally, we conclude our work in “Conclusion” section.

## Related work

Currently, there are three main research directions for device recognition: traffic-based device recognition methods, network-search-based device recognition methods, and physical-information-based device recognition methods.

In traffic-based device recognition methods, the authors mine and analyze the attribute information (such as ports, protocols, banners, etc.) in active measurement traffic, and the behavior features (such as packet length, sending interval, and statistical feature of data flow) in passive monitor traffic. After that, the authors build a device fingerprint database or train device-recognition classifiers to realize the discrimination of the device type. In Nmap^8^, the device’s network ports are extracted from the active measurement traffic, and the device fingerprint is calculated by those port results. Based on these device fingerprints, Nmap tool identifies the service type and operating system of device. After that, Durumeric et al.^9^ develop Zmap, which greatly accelerate the device information collection before recognition. So, Zmap improve the recognition efficiency of network devices. In paper^10–14^, the authors extract the IP, port, flag bits, and others in the header of the TCP packet as features, and use machine learning algorithms to train the recognition classifiers, thereby realizing the discrimination of the device type. Those methods in papers^15–18^ extract features from the protocol data of each layer from passive monitor traffic to form device fingerprints, and identify the type of target device by matching fingerprint. With slight differences in the previously mentioned methods, those methods in paper^19–21^ extract features (such as packet time, length, port, DNS protocol, etc.) from the network layer data and application layer data in traffic, and use a variety of machine learning algorithms to build a phased recognition classifier to identify device. Cheng et al.^22^ recognize the device according to the difference between the file headers of devices in active measurement traffic. To improve the security in training model, He et al.^23^ build a recognition model based on federal learning. To improve the usability of the recognition model, Jiao et al.^24^ propose a multi-level identification framework to decrease the updating frequency of recognition model when training data is updated. In addition, the methods in^25,26^ do not need to extract features from traffic and eliminates the influence of features extraction behavior during the recognition process. The traffic-based device recognition method can easily obtain the measurement data, and can identify device types and brands in batches in normal network environment. In^27^, the authors summarize the traffic-based device recognition methods. In real life, the systems, built based on this type of methods, such as Shodan^28^, ZoomEye^29^, Censys^30^, and Quake^31^ have been widely used. However, because same categories of devices with one brand often use same protocol to transmit data, the difference between these devices in traffic is not obvious. This kind of methods is difficult to effectively recognize fine-grained model of device.

In network-search-based device recognition methods, the authors use the Internet crawlers to acquire device information from Internet resources such as URL (Uniform Resource Locator) strings and Web pages, so as to construct a device database for device recognition. Li et al.^32^ implement a device recognition algorithm based on the GUI (Graphical User Interface) information in the web pages of camera devices, and found about 1.6 million camera devices. Zou et al.^33^ establish an IoT device recognition framework. In this framework, Zou et al. built a device database based on the devices’ attributes in the IoT device protocol slogan, and then realized the hierarchical recognition of device. Agarwal et al.^34^ develop a tool named WID to recognize the device from the source code of webpages and subpages of devices. ARE^35^ could search for special slogans in device webpage, and obtained the device description information from the device annotation to identify device. Those methods can recognize the model of device without building a device fingerprint database or training machine learning classifier. However, because the reliability of Web resources is difficult to evaluate and the webpages’ structure of search results is diverse, extracting reliable device information from webpages is complex. This kind of methods are not easy to implement, and the recognition accuracy of those methods is limited in practical work.

In physical-attributes-based device recognition methods, the authors recognize the type of device based on the difference in the physical characteristics. Guo et al.^36^ analyze the structure characteristics of device physical addresses and recognize the type of device based on the device’s MAC (Media Access Control) addresses. The methods in paper^37–43^ use the time offset characteristics of “the clock of each device is unique, and the deviation still exists after being calibrated by the NTP (Network Time Protocol)” to recognize IoT devices. Radhakrishnan^44^ found that the device hardware clock deviation would lead to differences in network behavior. So, Radhakrishnan designs a fingerprint generation algorithm using neural network, named GTID, to identify device types. The device recognition methods based on physical attributes can identify the model of device. Especially, the recognition methods based on the clock offset characteristic, greatly improve the recognition accuracy. However, due to the interference in network, precise time offset of device is difficult to obtain, causing low recognition accuracy of these methods in the actual network. At the same time, measuring the device’s clock offset is not easy, which also limits the widely application of these methods.

In this paper, we extract traffic attributes such as GPU model, resolution and operating system, as well as the physical attributes such as device size and screen size, and propose Recognizer, a high accuracy model recognition method of mobile device based on weighted feature similarity. Recognizer extracts the common attributes of all mobile devices as features, and formulates feature similarity calculation rules according to feature expression, so as to measure the similarity between different devices. At the same time, we design the features importance evaluation strategies to assess the role of each feature in brand recognition (we call this strategy “RFBR”) and model recognition (we call this strategy “RFMR”). In RFBR and RFMR, the weight of features will be determined. When the target device recognition is performed, brand recognition and model recognition are performed in sequence, so as to obtain the model of target device.

## Our method: Recognizer

In this section, we will introduce the principles and steps of the Recognizer in detail.

### Symbol description

*f*: feature. There are three kinds of feature: extracted feature *f*_e_, brand feature *f*_b_ and model feature *f*_m_. Among them, *f*_e_ is the feature extracted from the public attributes of the device, *f*_b_ is the brand feature selected from the extracted features using the RFBR algorithm, and *f*_m_ is the model feature selected from the extracted features using the RFMR algorithm. The general representations of the *i*th extracted feature, brand feature, and model feature are $f_{{\text{e}}}^{{\left( { * ,i} \right)}}$, $f_{{\text{b}}}^{{\left( { * ,i} \right)}}$, and $f_{{\text{m}}}^{{\left( { * ,i} \right)}}$. For the device *D*_*i*_, the *i*th extracted feature, *i*th brand feature and *i*th model feature are denoted as $f_{{\text{e}}}^{{\left( {D_{i} ,i} \right)}}$, $f_{{\text{b}}}^{{\left( {D_{i} ,i} \right)}}$ and $f_{{\text{m}}}^{{\left( {D_{i} ,i} \right)}}$ respectively.

**F**: feature vector. There are three kinds of feature vector: extraction feature vector **F**_e_, brand feature vector **F**_b_ and model feature vector **F**_m_. Among them, **F**_e_ is a vector composed of extracted features *f*_e_, ${\mathbf{F}}_{{\text{e}}} = \left[ {f_{{\text{e}}}^{{\left( { * ,1} \right)}} ,f_{{\text{e}}}^{{\left( { * ,2} \right)}} , \ldots } \right]$. **F**_b_ is a vector composed of brand features *f*_b_, ${\mathbf{F}}_{{\text{b}}} = \left[ {f_{{\text{b}}}^{{\left( { * ,1} \right)}} ,f_{{\text{b}}}^{{\left( { * ,2} \right)}} , \ldots } \right]$. **F**_m_ is a vector composed of model features *f*_m_, ${\mathbf{F}}_{{\text{m}}} = \left[ {f_{{\text{m}}}^{{\left( { * ,1} \right)}} ,f_{{\text{m}}}^{{\left( { * ,2} \right)}} , \ldots } \right]$. For the device *D*_*i*_, the *i*th extracted feature vector, *i*th brand feature vector and *i*th model feature vector are denoted as ${\mathbf{F}}_{{\text{e}}}^{{\left( {D_{i} } \right)}}$ (${\mathbf{F}}_{{\text{e}}}^{{\left( {D_{i} } \right)}} = \left[ {f_{{\text{e}}}^{{\left( {D_{i} ,1} \right)}} ,f_{{\text{e}}}^{{\left( {D_{i} ,2} \right)}} , \ldots } \right]$), ${\mathbf{F}}_{{\text{b}}}^{{\left( {D_{i} } \right)}}$ (${\mathbf{F}}_{{\text{b}}}^{{\left( {D_{i} } \right)}} = \left[ {f_{{\text{b}}}^{{\left( {D_{i} ,1} \right)}} ,f_{{\text{b}}}^{{\left( {D_{i} ,2} \right)}} , \ldots } \right]$) and ${\mathbf{F}}_{{\text{m}}}^{{\left( {D_{i} } \right)}}$ (${\mathbf{F}}_{{\text{m}}}^{{\left( {D_{i} } \right)}} = \left[ {f_{{\text{m}}}^{{\left( {D_{i} ,1} \right)}} ,f_{{\text{m}}}^{{\left( {D_{i} ,2} \right)}} , \ldots } \right]$) respectively.

$S\left( {f^{{\left( {D_{i} ,k} \right)}} ,f^{{\left( {D_{j} ,k} \right)}} } \right)$: similarity function between two device features, $0 \le S\left( {f^{{\left( {D_{i} ,k} \right)}} ,f^{{\left( {D_{j} ,k} \right)}} } \right) \le 1$. The similarity functions of the *k*th extracted feature, brand feature and model feature of device *D*_*i*_ and *D*_*j*_ are denoted as $S\left( {f_{{\text{e}}}^{{\left( {D_{i} ,k} \right)}} ,f_{{\text{e}}}^{{\left( {D_{j} ,k} \right)}} } \right)$, $S\left( {f_{{\text{b}}}^{{\left( {D_{i} ,k} \right)}} ,f_{{\text{b}}}^{{\left( {D_{j} ,k} \right)}} } \right)$ and $S\left( {f_{{\text{m}}}^{{\left( {D_{i} ,k} \right)}} ,f_{{\text{m}}}^{{\left( {D_{j} ,k} \right)}} } \right)$.

${\mathbf{S}}\left( {{\mathbf{F}}^{{\left( {D_{i} } \right)}} ,{\mathbf{F}}^{{\left( {D_{i} } \right)}} } \right)$: similarity function vector. ${\mathbf{S}}\left( {{\mathbf{F}}^{{\left( {D_{i} } \right)}} ,{\mathbf{F}}^{{\left( {D_{i} } \right)}} } \right) = \left[ {S\left( {f^{{\left( {D_{i} ,1} \right)}} ,f^{{\left( {D_{j} ,1} \right)}} } \right),S\left( {f^{{\left( {D_{i} ,2} \right)}} ,f^{{\left( {D_{j} ,2} \right)}} } \right), \ldots } \right]$. The similarity function vectors of the extracted feature vector, brand feature vector and model feature vector of device *D*_*i*_ and *D*_*j*_ are denoted as ${\mathbf{S}}\left( {{\mathbf{F}}_{{\text{e}}}^{{\left( {D_{i} } \right)}} ,{\mathbf{F}}_{{\text{e}}}^{{\left( {D_{j} } \right)}} } \right)$, ${\mathbf{S}}\left( {{\mathbf{F}}_{{\text{b}}}^{{\left( {D_{i} } \right)}} ,{\mathbf{F}}_{{\text{b}}}^{{\left( {D_{j} } \right)}} } \right)$ and ${\mathbf{S}}\left( {{\mathbf{F}}_{{\text{m}}}^{{\left( {D_{i} } \right)}} ,{\mathbf{F}}_{{\text{m}}}^{{\left( {D_{j} } \right)}} } \right)$.

${\mathbf{F}}\backslash f^{{\left( { * ,i} \right)}}$: result of removing $f^{{\left( { * ,i} \right)}}$ from the feature vector **F**. For the extracted feature vector ${\mathbf{F}}_{{\text{e}}}^{{\left( {D_{i} } \right)}}$ of device *D*_*i*_, if ${\mathbf{F}}_{{\text{e}}}^{{\left( {D_{i} } \right)}} = \left[ {f_{{\text{e}}}^{{\left( {D_{i} ,1} \right)}} ,f_{{\text{e}}}^{{\left( {D_{i} ,2} \right)}} ,f_{{\text{e}}}^{{\left( {D_{i} ,3} \right)}} } \right]$, then ${\mathbf{F}}_{{\text{e}}}^{{\left( {D_{i} } \right)}} \backslash f_{{\text{e}}}^{{\left( {D_{i} ,2} \right)}} = \left[ {f_{{\text{e}}}^{{\left( {D_{i} ,1} \right)}} ,f_{{\text{e}}}^{{\left( {D_{i} ,3} \right)}} } \right]$.

*B*_a_: collection of all devices whose brand is a, $B_{{\text{a}}} = \left\{ {D_{1} ,D_{2} , \ldots } \right\}$. $\left| {B_{{\text{a}}} } \right|$ is the number of elements in *B*_a_.

***B***: Collection of all device brands, ${\mathbf{B}} = \left\{ {B_{{\text{a}}} ,B_{{\text{b}}} , \ldots } \right\}$. *M* is the size of ***B***, $M = \left| {\mathbf{B}} \right|$.

${\mathbf{B}} - \left\{ {B_{i} } \right\}$: result of removing *B*_*i*_ from ***B***. if ${\mathbf{B}} = \left\{ {B_{{\text{a}}} ,B_{{\text{b}}} ,B_{{\text{c}}} } \right\}$, then ${\mathbf{B}} - \left\{ {B_{{\text{b}}} } \right\} = \left\{ {B_{{\text{a}}} ,B_{{\text{c}}} } \right\}$.

$\overrightarrow {(t)}$: this is a *t*-dimensional row vector, and each value in the vector is 1/*t*. For example, $\overrightarrow {(2)} = [0.5,0.5]$.

$$\min \left| {(a - b),\varepsilon } \right|$$: minimum of $$\left| {a - b} \right|$$, $$\left| {a - b + \varepsilon } \right|$$, $$\left| {a - b - \varepsilon } \right|$$.

### Principles and steps of Recognizer

**Figure 1:**
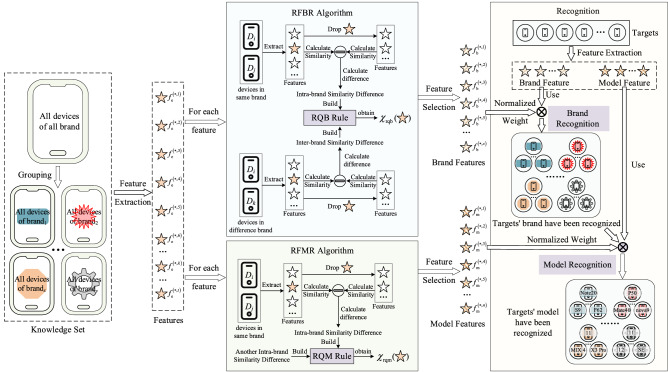
The framework of Recognizer.

Recognizer first extracts the common attributes of all mobile devices as features, and formulates similarity calculation rules according to the expression of extracted features. Then, we propose RFBR and RBMR strategies to assess the role of each feature in brand recognition and model recognition for feature selection and weight determination. Finally, Recognizer uses the target’s features to identify the brand and model. The framework of Recognizer is shown in Fig. 1.

There are 7 steps in Recognizer, as follows:

- *Step 1* Group devices. All devices in the knowledge set are grouped by device brand. In each group, all devices’ brands are same.
- *Step 2* Extract feature. In each group, we extract the common attributes of all devices as brand attributes. If all devices in all groups own one attribute, this attribute will be as a feature.
- *Step 3* Calculate similarity between two features. According to the form of extracted feature, we divide the extracted features into numerical features and string features. For each feature form, we build the feature similarity calculation strategy.
- *Step 4* Select brand feature. Based on the effect of each feature on the similarity between same-brand devices and the similarity between devices with different brands, we propose RFBR strategy to quantify the importance of each feature in brand recognition, and the importance value is expressed as ${\chi }_{\mathrm{rqb}}$. Those features, whose ${\chi }_{\mathrm{rqb}}$ is greater than 0, will be selected as brand features. And the value of ${\chi }_{\mathrm{rqb}}$ is as the weight of brand feature.
- *Step 5* Select model feature. Because one model only corresponds to one mobile device, there is no similarity between devices with same model. So, it is unreasonable to use RFBR strategy for model feature selection. According to the effect of feature on same-brand devices and the difference of effect on all brands, we propose RFMR strategy to quantify the importance of each feature in model recognition, and the importance value is expressed as ${\chi }_{\mathrm{rqm}}$. Those features, whose ${\chi }_{\mathrm{rqm}}$ is greater than 0, are selected as model feature. And ${\chi }_{\mathrm{rqm}}$ is the weight of feature.
- *Step 6* Normalize weights. All weights of brand features obtained in Step 4 and all weights of model features obtained in Step 5 are normalized respectively.
- *Step 7* Recognize target’s model. We obtain brand features and model features from target mobile device. After recognizing the brand of target according to brand features and brand features’ weights, the model features and model features’ weights are used to identify the model of target.

### Key steps of Recognizer

Among all steps of Recognizer, Step 3, 4, 5, 7 are key steps. These key steps are described in detail as follows.

1. Calculate similarity between two features.

We divide the extracted features into numerical features and string features. For a feature, if the feature value is a numeric value obtained by measurement tool and there is an inevitable measurable error due to the precision limitation of the measurement tool, the feature is a numeric feature (e.g., length); otherwise, the feature is a string feature (e.g., operating system).

We measure the similarity between two numerical features based on the difference value between two values, while the similarity between two string features is determined based on the inclusion relationship between two strings. Certainly, although a number can be considered as a string, it is not reasonable to calculate the similarity between two numerical features based on the inclusion relationship. For example, for two numeric features *f*_1_ (value is 1000) and *f*_2_ (value is 999), if *f*_1_ and *f*_2_ are regarded as string features, the similarity value between *f*_1_ and *f*_2_ is 0. Obviously, it is unreasonable. Therefore, for two types of features, we design two strategies to calculate the similarity between features, respectively, as follows.

- Numerical feature similarity strategy

For numerical features, the smaller the difference in two feature values, the more similar the two features are. But, due to the error in measurement, there is a deviation between the measurement value and the actual value. So, considering the measurement error in numerical feature similarity strategy is more reasonable, which could reduce the effect of measurement error when calculating similarity between two numerical features. According to this, we define (1) and (2) as numerical feature similarity calculation rules.

If the *k*th extracted features of base device *D*_*i*_ and target device *D*_*j*_ are one-dimensional numerical features, the similarity between $f_{{\text{e}}}^{{\left( {D_{j} ,k} \right)}}$ and $f_{{\text{e}}}^{{\left( {D_{i} ,k} \right)}}$ is calculated by (1).

1 $$S\left( {f_{{\text{e}}}^{{\left( {D_{i} ,k} \right)}} ,f_{{\text{e}}}^{{\left( {D_{j} ,k} \right)}} } \right) = \left\{ {\begin{array}{*{20}l} { - \frac{{\min \left| {\left( {\left| {f_{{\text{e}}}^{{\left( {D_{i} ,k} \right)}} } \right| - \left| {f_{{\text{e}}}^{{\left( {D_{j} ,k} \right)}} } \right|} \right),\varepsilon } \right|}}{{f_{{\text{e}}}^{{\left( {D_{i} ,k} \right)}} }},} \hfill & {\left| {f_{{\text{e}}}^{{\left( {D_{j} ,k} \right)}} } \right| \le 2\left| {f_{{\text{e}}}^{{\left( {D_{i} ,k} \right)}} } \right|} \hfill \\ {0,} \hfill & {else} \hfill \\ \end{array} } \right.$$

In (1), $\varepsilon$ is the measurement error threshold, and $\left| {f_{{\text{e}}}^{{\left( {D_{i} ,k} \right)}} } \right|$ is the absolute value of numerical feature.

If $f_{{\text{e}}}^{{\left( {D_{j} ,k} \right)}}$ and $f_{{\text{e}}}^{{\left( {D_{i} ,k} \right)}}$ are multi-dimensional numerical features, the dimensional similarity is calculated for the values in each dimension, and the feature similarity is the product of all dimensional similarities. If $f_{{\text{e}}}^{{\left( {D_{i} ,k} \right)}} = (v_{k,1}^{{\left( {D_{i} } \right)}} , \ldots ,v_{k,s}^{{\left( {D_{i} } \right)}} )$ and $f_{{\text{e}}}^{{\left( {D_{j} ,k} \right)}} = (v_{k,1}^{{\left( {D_{j} } \right)}} , \ldots v_{k,s}^{{\left( {D_{j} } \right)}} )$, the similarity between the target feature $f_{{\text{e}}}^{{\left( {D_{j} ,k} \right)}}$ and base feature $f_{{\text{e}}}^{{\left( {D_{i} ,k} \right)}}$ is calculated using (2).

2 $$S\left( {f_{{\text{e}}}^{{\left( {D_{i} ,k} \right)}} ,f_{{\text{e}}}^{{\left( {D_{j} ,k} \right)}} } \right) = \prod\limits_{t = 1}^{s} {S\left( {v_{k,t}^{{\left( {D_{i} } \right)}} ,v_{k,t}^{{\left( {D_{j} } \right)}} } \right)}$$

When calculating the feature similarity according to (1) and (2), if $\left| {f_{{\text{e}}}^{{\left( {D_{j} ,k} \right)}} } \right| > 2\left| {f_{{\text{e}}}^{{\left( {D_{i} ,k} \right)}} } \right|$ or $\left| {v_{k,t}^{{\left( {D_{j} } \right)}} } \right| > 2\left| {v_{k,t}^{{\left( {D_{i} } \right)}} } \right|$, it indicates that the difference between two numerical feature values (or two values in a certain dimension) is too large. In this case, we think that the two numerical features are not similar, the feature similarity value is set as 0.

(b) String feature similarity strategy

Since each string represents a specific meaning, each string is considered as a whole to calculate the similarity. In Recognizer, according to the number of strings in string feature, the string features are divided into single-string feature and multi-strings feature. We define (3) and (4) as string feature similarity rules.

If the *k*th extracted features $f_{{\text{e}}}^{{\left( {D_{i} ,k} \right)}}$ and $f_{{\text{e}}}^{{\left( {D_{j} ,k} \right)}}$ of devices *D*_*i*_ and *D*_*j*_ are single-string features, we calculate the feature similarity between $f_{{\text{e}}}^{{\left( {D_{j} ,k} \right)}}$ and $f_{{\text{e}}}^{{\left( {D_{i} ,k} \right)}}$ according to (3).

3 $$S\left( {f_{{\text{e}}}^{{\left( {D_{i} ,k} \right)}} ,f_{{\text{e}}}^{{\left( {D_{j} ,k} \right)}} } \right) = \left\{ {\begin{array}{*{20}l} {1,} \hfill & {f_{{\text{e}}}^{{\left( {D_{j} ,k} \right)}} = f_{{\text{e}}}^{{\left( {D_{i} ,k} \right)}} } \hfill \\ {0.8,} \hfill & { \, f_{{\text{e}}}^{{\left( {D_{j} ,k} \right)}} \in f_{{\text{e}}}^{{\left( {D_{i} ,k} \right)}} \, or \, f_{{\text{e}}}^{{\left( {D_{i} ,k} \right)}} \in f_{{\text{e}}}^{{\left( {D_{j} ,k} \right)}} } \hfill \\ {0,} \hfill & {else} \hfill \\ \end{array} } \right.$$

When $f_{{\text{e}}}^{{\left( {D_{j} ,k} \right)}} \in f_{{\text{e}}}^{{\left( {D_{i} ,k} \right)}}$ or $f_{{\text{e}}}^{{\left( {D_{i} ,k} \right)}} \in f_{{\text{e}}}^{{\left( {D_{j} ,k} \right)}}$, we think that the feature value is incomplete. In this case, the feature similarity value is set to 0.8 (this is an experience value).

If $f_{{\text{e}}}^{{\left( {D_{i} ,k} \right)}}$ and $f_{{\text{e}}}^{{\left( {D_{j} ,k} \right)}}$ are multi-strings features, we construct vector space with $f_{{\text{e}}}^{{\left( {D_{i} ,k} \right)}} \cup f_{{\text{e}}}^{{\left( {D_{j} ,k} \right)}}$, and vectorize $f_{{\text{e}}}^{{\left( {D_{j} ,k} \right)}}$ and $f_{{\text{e}}}^{{\left( {D_{i} ,k} \right)}}$. At this time, the cosine similarity between two vectors is the similarity between two multi-strings features. For example, if $f_{{\text{e}}}^{{\left( {D_{i} ,k} \right)}} = \left\{ {str1,str2} \right\}$ and $f_{{\text{e}}}^{{\left( {D_{j} ,k} \right)}} = \left\{ {str2,str3} \right\}$, the vector space is $\left\{ {str1,str2,str3} \right\}$. At this time, the vectorization result of $f_{{\text{e}}}^{{\left( {D_{i} ,k} \right)}}$ is $[1,1,0]$, and the vectorization result of $f_{{\text{e}}}^{{\left( {D_{j} ,k} \right)}}$ is [0, 1, 1]. We denote the vectorization results of $f_{{\text{e}}}^{{\left( {D_{j} ,k} \right)}}$ and $f_{{\text{e}}}^{{\left( {D_{i} ,k} \right)}}$ as $V_{j,k}$ and $V_{i,k}$ respectively, then the feature similarity between $f_{{\text{e}}}^{{\left( {D_{j} ,k} \right)}}$ and $f_{{\text{e}}}^{{\left( {D_{i} ,k} \right)}}$ is calculated by (4).

4 $$S\left( {f_{{\text{e}}}^{{\left( {D_{i} ,k} \right)}} ,f_{{\text{e}}}^{{\left( {D_{j} ,k} \right)}} } \right) = \frac{{V_{i,k} \cdot V_{j,k} }}{{\left| {V_{i,k} } \right| \cdot \left| {V_{j,k} } \right|}}$$

(2) Select brand feature

In Recognizer, we use RFBR strategy for brand feature selection. So, we describe RFBR in detail here.

Assuming that $f_{{\text{e}}}^{{\left( { * ,1} \right)}} ,f_{{\text{e}}}^{{\left( { * ,2} \right)}} ,f_{{\text{e}}}^{{\left( { * ,3} \right)}} , \ldots ,f_{{\text{e}}}^{{\left( { * ,n} \right)}}$ are all extraction features, then extract the feature vector ${\mathbf{F}}_{{\text{e}}} = [f_{{\text{e}}}^{{\left( { * ,1} \right)}} ,f_{{\text{e}}}^{{\left( { * ,2} \right)}} ,f_{{\text{e}}}^{{\left( { * ,3} \right)}} , \ldots ,f_{{\text{e}}}^{{\left( { * ,n} \right)}} ]$. For each extracted feature $f_{{\text{e}}}^{{\left( { * ,m} \right)}}$, $1 \le m \le n$, ${\mathbf{F}}_{{\text{e}}}^{\prime } = {\mathbf{F}}_{{\text{e}}} \backslash f_{{\text{e}}}^{{\left( { * ,m} \right)}}$, we calculate the mean of intra-brand similarity increments according to (5).

5 $$\varphi \left( {f_{{\text{e}}}^{{\left( { * ,m} \right)}} } \right) = \frac{1}{M}\sum\limits_{k} {\frac{{\sum\limits_{i} {\sum\limits_{j} {{\mathbf{S}}\left( {{\mathbf{F}}_{{\text{e}}}^{{\left( {D_{i} } \right)}} ,{\mathbf{F}}_{{\text{e}}}^{{\left( {D_{j} } \right)}} } \right)\overrightarrow {\left( n \right)}^{{\varvec{T}}} - {\mathbf{S}}\left( {{\mathbf{F}}_{{\text{e}}}^{{\left( {D_{i} } \right)\prime }} ,{\mathbf{F}}_{{\text{e}}}^{{\left( {D_{j} } \right)\prime }} } \right)\overrightarrow {{\left( {n - 1} \right)}}^{{\varvec{T}}} } } }}{{\left| {B_{k} } \right|^{2} }}}$$

In (5), $D_{i} ,D_{j} \in B_{k}$, $B_{k} \in {\varvec{B}}$, ${\mathbf{F}}_{{\text{e}}}^{{\left( {D_{i} } \right)\prime }} = {\mathbf{F}}_{{\text{e}}}^{{\left( {D_{i} } \right)}} \backslash f_{{\text{e}}}^{{\left( {D_{i} ,m} \right)}}$, and ${\mathbf{F}}_{{\text{e}}}^{{\left( {D_{j} } \right)\prime }} = {\mathbf{F}}_{{\text{e}}}^{{\left( {D_{j} } \right)}} \backslash f_{{\text{e}}}^{{\left( {D_{j} ,m} \right)}}$. Meanwhile, we calculate the mean of inter-brand similarity increments according to (6).

6 $$\delta \left( {f_{{\text{e}}}^{{\left( { * ,m} \right)}} } \right) = \frac{1}{M}\sum\limits_{k} {\frac{{\sum\limits_{l} {\sum\limits_{i} {{\mathbf{S}}\left( {{\mathbf{F}}_{{\text{e}}}^{{\left( {D_{i} } \right)}} ,{\mathbf{F}}_{{\text{e}}}^{{\left( {D_{l} } \right)}} } \right)\overrightarrow {\left( n \right)}^{{\varvec{T}}} - {\mathbf{S}}\left( {{\mathbf{F}}_{{\text{e}}}^{{\left( {D_{i} } \right)\prime }} ,{\mathbf{F}}_{{\text{e}}}^{{\left( {D_{l} } \right)\prime }} } \right)\overrightarrow {{\left( {n - 1} \right)}}^{{\varvec{T}}} } } }}{{\left| {B_{k} } \right|\left| {{\varvec{B}} - \left\{ {B_{k} } \right\}} \right|}}}$$

In (6), $D_{i} \in B_{k}$, $B_{k} \in {\mathbf{B}}$, $D_{l} \in {\varvec{B}} - \left\{ {B_{k} } \right\}$, ${\mathbf{F}}_{{\text{e}}}^{{\left( {D_{i} } \right)\prime }} = {\mathbf{F}}_{{\text{e}}}^{{\left( {D_{i} } \right)}} \backslash f_{{\text{e}}}^{{\left( {D_{i} ,m} \right)}}$, and ${\mathbf{F}}_{{\text{e}}}^{{\left( {D_{l} } \right)\prime }} = {\mathbf{F}}_{{\text{e}}}^{{\left( {D_{l} } \right)}} \backslash f_{{\text{e}}}^{{\left( {D_{l} ,m} \right)}}$. Due to $0 \le S(f^{{\left( {D_{i} ,k} \right)}} ,f^{{\left( {D_{j} ,k} \right)}} ) \le 1$, according to (5) and (6), we can obtain (7).

7 $$- 1 \le \varphi \left( {f_{{\text{e}}}^{{\left( { * ,m} \right)}} } \right),\delta \left( {f_{{\text{e}}}^{{\left( { * ,m} \right)}} } \right) \le 1$$

According to $\varphi \left( {f_{{\text{e}}}^{{\left( { * ,m} \right)}} } \right)$ and $\delta \left( {f_{{\text{e}}}^{{\left( { * ,m} \right)}} } \right)$, we design a rule (we name it RQB rule, as 8) to quantifying the feature-differentiation in brand recognition to assess the role of $f_{{\text{e}}}^{{\left( { * ,m} \right)}}$ in brand recognition.

8 $$\chi_{{{\text{rqb}}}} \left( {f_{{\text{e}}}^{{\left( { * ,m} \right)}} } \right) = \left\{ {\begin{array}{*{20}l} {\alpha \varphi \left( {f_{{\text{e}}}^{{\left( { * ,m} \right)}} } \right) - \left( {1 - \alpha } \right)\delta \left( {f_{{\text{e}}}^{{\left( { * ,m} \right)}} } \right),} \hfill & {\varphi \left( {f_{{\text{e}}}^{{\left( { * ,m} \right)}} } \right) \ge 0} \hfill \\ {0,} \hfill & {\varphi \left( {f_{{\text{e}}}^{{\left( { * ,m} \right)}} } \right) < 0} \hfill \\ \end{array} } \right.$$

In (8), $\alpha$ is an adjustable parameter, $\alpha \in [0,1]$. If $\chi_{{{\text{rqb}}}} (f_{{\text{e}}}^{{\left( { * ,m} \right)}} ) > 0$, $f_{{\text{e}}}^{{\left( { * ,m} \right)}}$ will be selected as the brand feature, and the weight of $f_{{\text{e}}}^{{\left( { * ,m} \right)}}$ is $\chi_{{{\text{rqb}}}} (f_{{\text{e}}}^{{\left( { * ,m} \right)}} )$.

(3) Select model feature

In Recognizer, we use RFMR for model feature selection. So, we describe the process of RFMR in detail here.

Assuming that $f_{{\text{e}}}^{{\left( { * ,1} \right)}} ,f_{{\text{e}}}^{{\left( { * ,2} \right)}} ,f_{{\text{e}}}^{{\left( { * ,3} \right)}} , \ldots ,f_{{\text{e}}}^{{\left( { * ,n} \right)}}$ are all extraction features, then extract the feature vector ${\mathbf{F}}_{{\text{e}}} = [f_{{\text{e}}}^{{\left( { * ,1} \right)}} ,f_{{\text{e}}}^{{\left( { * ,2} \right)}} ,f_{{\text{e}}}^{{\left( { * ,3} \right)}} , \ldots f_{{\text{e}}}^{{\left( { * ,n} \right)}} ]$. For each extracted feature $f_{{\text{e}}}^{{\left( { * ,m} \right)}}$, $1 \le m \le n$, ${\mathbf{F}}_{{\text{e}}}^{\prime } = {\mathbf{F}}_{{\text{e}}} \backslash f_{{\text{e}}}^{{\left( { * ,m} \right)}}$, we calculate $\varphi \left( {f_{{\text{e}}}^{{\left( { * ,m} \right)}} } \right)$ according to (5), and calculate the incremental standard deviation of intra-brand similarity according to formula (9).

9 $$\left\{ {\begin{array}{*{20}l} {E\left( {B_{k} } \right) = \frac{{\sum\limits_{i} {\sum\limits_{j} {{\mathbf{S}}\left( {{\mathbf{F}}_{{\text{e}}}^{{\left( {D_{i} } \right)}} ,{\mathbf{F}}_{{\text{e}}}^{{\left( {D_{j} } \right)}} } \right)\overrightarrow {\left( n \right)}^{\mathbf{T}} - {\mathbf{S}}\left( {{\mathbf{F}}_{{\text{e}}}^{{\left( {D_{i} } \right)\prime }} ,{\mathbf{F}}_{{\text{e}}}^{{\left( {D_{j} } \right)\prime }} } \right)\overrightarrow {{\left( {n - 1} \right)}}^{\mathbf{T}} } } }}{{\left| {B_{k} } \right|^{2} }}} \hfill \\ {\gamma \left( {f_{{\text{e}}}^{{\left( { * ,m} \right)}} } \right) = \frac{1}{M}\sum\limits_{k} {\left( {\frac{{\sum\limits_{i} {\sum\limits_{j} {\left( {{\mathbf{S}}\left( {{\mathbf{F}}_{{\text{e}}}^{{\left( {D_{i} } \right)}} ,{\mathbf{F}}_{{\text{e}}}^{{\left( {D_{j} } \right)}} } \right)\overrightarrow {\left( n \right)}^{\mathbf{T}} - {\mathbf{S}}\left( {{\mathbf{F}}_{{\text{e}}}^{{\left( {D_{i} } \right)\prime }} ,{\mathbf{F}}_{{\text{e}}}^{{\left( {D_{j} } \right)\prime }} } \right)\overrightarrow {{\left( {n - 1} \right)}}^{\mathbf{T}} - E\left( {B_{k} } \right)} \right)^{2} } } }}{{\left| {B_{k} } \right|^{2} }}} \right)^{\frac{1}{2}} } } \hfill \\ \end{array} } \right.$$

In (9), $$D_{i} ,D_{j} \in B_{k}$$, $$B_{k} \in {\mathbf{B}}$$, $${\mathbf{F}}_{{\text{e}}}^{{\left( {D_{i} } \right)\prime }} = {\mathbf{F}}_{{\text{e}}}^{{\left( {D_{i} } \right)}} \backslash f_{{\text{e}}}^{{\left( {D_{i} ,m} \right)}}$$, $${\mathbf{F}}_{{\text{e}}}^{{\left( {D_{j} } \right)\prime }} = {\mathbf{F}}_{{\text{e}}}^{{\left( {D_{j} } \right)}} \backslash f_{{\text{e}}}^{{\left( {D_{j} ,m} \right)}}$$.

According to $\varphi \left( {f_{{\text{e}}}^{{\left( { * ,m} \right)}} } \right)$ and $\gamma \left( {f_{{\text{e}}}^{{\left( { * ,m} \right)}} } \right)$, we design a rule (we name it RQM rule, as 10) to quantifying the feature-differentiation in model recognition to assess the role of $f_{{\text{e}}}^{{\left( { * ,m} \right)}}$ in model recognition.

10 $$\chi_{{{\text{rqm}}}} \left( {f_{{\text{e}}}^{{\left( { * ,m} \right)}} } \right) = \left\{ {\begin{array}{*{20}l} {0,} \hfill & {\varphi \left( {f_{{\text{e}}}^{{\left( { * ,m} \right)}} } \right) \ge 0} \hfill \\ { - \beta \varphi \left( {f_{{\text{e}}}^{{\left( { * ,m} \right)}} } \right) - \left( {1 - \beta } \right)\gamma \left( {f_{{\text{e}}}^{{\left( { * ,m} \right)}} } \right),} \hfill & {\varphi \left( {f_{{\text{e}}}^{{\left( { * ,m} \right)}} } \right) < 0} \hfill \\ \end{array} } \right.$$

In (10), $\beta$ is an adjustable parameter, $\beta \in [0.5,1]$. If $\chi_{{{\text{rqm}}}} (f_{{\text{e}}}^{{\left( { * ,m} \right)}} ) > 0$, $f_{{\text{e}}}^{{\left( { * ,m} \right)}}$ will be selected as the model feature, and the weight of $f_{{\text{e}}}^{{\left( { * ,m} \right)}}$ is $\chi_{{{\text{rqm}}}} (f_{{\text{e}}}^{{\left( { * ,m} \right)}} )$.

(4) Recognize device type

In device type recognition, there are two parts: brand recognition and model recognition. We first perform brand recognition on the target device, and then perform model recognition.

In brand recognition, brand features and normalized weights are used in (11) to calculate the similarity between target device and known devices.

11 $$\Phi \left( {K_{i} ,T} \right) = {\mathbf{S}}\left( {{\mathbf{F}}_{{\text{b}}}^{{\left( {K_{i} } \right)}} ,{\mathbf{F}}_{{\text{b}}}^{\left( T \right)} } \right){\mathbf{W}}\left( {{\mathbf{F}}_{{\text{b}}} } \right)$$

In (11), *T* is the target device, *K*_*i*_ is one known device in the knowledge set, and ${\mathbf{W}}\left( {{\mathbf{F}}_{{\text{b}}} } \right)$ is the standardized weight vector of brand feature. In knowledge set, the brand of known device with the greatest similarity with target device is taken as the brand of target device. So as to realize brand recognition of the target device.

In model recognition, model features and normalized weights are used in (12) to calculate the similarity between target device and known devices. At this time, the brand of known devices is same with target device.

12 $$\Psi \left( {K_{i} ,T} \right) = {\mathbf{S}}\left( {{\mathbf{F}}_{{\text{m}}}^{{\left( {K_{i} } \right)}} ,{\mathbf{F}}_{{\text{m}}}^{\left( T \right)} } \right){\mathbf{W}}\left( {{\mathbf{F}}_{{\text{m}}} } \right)$$

In (12), *T* is the target device, *K*_*i*_ is one known device in the knowledge set (the brand of *K*_*i*_ is same with target device), and ${\mathbf{W}}\left( {{\mathbf{F}}_{{\text{m}}} } \right)$ is the standardized weight vector of model feature. The model of known device with the greatest similarity with target device is taken as the model of target device. So as to realize model recognition of the target device.

## Analysis of Recognizer

In Recognizer, the brand features, model features and weights directly affect the accuracy of the recognition. We select brand features, model features and obtain their weights based on RFBR and RFMR strategies. Thus, in this section, we will analyze the rationality of RFBR and RFMR strategies.

### Rationality analysis of RFBR strategy

RFBR strategy is used to quantify the importance of extracted features in brand recognition, and to select brand features and determine weights.

According to the research of Fu et al.^45^, the judgment criterion, that the selected feature is effective, is that the selected features can increase the difference between classes (we denote this criterion as effective feature selection criterion, abbreviated as EFS criterion). Therefore, in Recognizer, if one extracted feature could be selected as a brand feature, this extracted feature should be able to increase the difference between devices with different brands. In brand feature selection, there are two cases meeting EFS criterion: (1) For one extracted feature, if the feature can increase the similarity between devices with same brand and reduce the similarity between those devices with different brands, this extracted feature will be selected as brand feature. This is the optimal case. (2) For one extracted feature, if the feature can simultaneously increase the similarity between devices with same brand and similarity between those devices with different brands, and the ratio, between inter-brand-similarity increments and intra-brand-similarity increments, is less than threshold, this extracted feature will also be selected as brand feature. This is an acceptable case.

Assuming that $f_{{\text{e}}}^{{\left( { * ,1} \right)}} ,f_{{\text{e}}}^{{\left( { * ,2} \right)}} ,f_{{\text{e}}}^{{\left( { * ,3} \right)}} , \ldots ,f_{{\text{e}}}^{{\left( { * ,n} \right)}}$ are all extraction features, then extract the feature vector ${\mathbf{F}}_{{\text{e}}} = [f_{{\text{e}}}^{{\left( { * ,1} \right)}} ,f_{{\text{e}}}^{{\left( { * ,2} \right)}} , \ldots ,f_{{\text{e}}}^{{\left( { * ,n} \right)}} ]$. $\forall D_{i} ,D_{j} \in B_{k} ,B_{k} \in {\mathbf{B}}$, $\forall D_{l} \in {\mathbf{B}} - \left\{ {B_{k} } \right\}$, ${\varvec{F}}_{{\text{e}}}^{{\left( {D_{i} } \right)\prime }} = {\varvec{F}}_{{\text{e}}}^{{\left( {D_{i} } \right)}} \backslash f_{{\text{e}}}^{{\left( {D_{i} ,m} \right)}}$, ${\varvec{F}}_{{\text{e}}}^{{\left( {D_{j} } \right)\prime }} = {\varvec{F}}_{{\text{e}}}^{{\left( {D_{j} } \right)}} \backslash f_{{\text{e}}}^{{\left( {D_{j} ,m} \right)}}$, ${\varvec{F}}_{{\text{e}}}^{{\left( {D_{l} } \right)\prime }} = {\varvec{F}}_{{\text{e}}}^{{\left( {D_{l} } \right)}} \backslash f_{{\text{e}}}^{{\left( {D_{l} ,m} \right)}}$. For each extracted feature $f_{{\text{e}}}^{{\left( { * ,m} \right)}}$ ($1 \le m \le n$), the similarity increment $s(f_{{\text{e}}}^{{\left( { * ,m} \right)}} )$ between any two devices with same brand and the similarity increment $d(f_{{\text{e}}}^{{\left( { * ,m} \right)}} )$ between any two devices with different brands are shown in (13).

13 $$\left\{ \begin{gathered} s\left( {f_{{\text{e}}}^{{\left( { * ,m} \right)}} } \right) = {\varvec{S}}\left( {{\varvec{F}}_{{\text{e}}}^{{\left( {D_{i} } \right)}} ,{\varvec{F}}_{{\text{e}}}^{{\left( {D_{j} } \right)}} } \right)\overrightarrow {\left( n \right)}^{\mathbf{T}} - {\varvec{S}}\left( {{\varvec{F}}_{{\text{e}}}^{{\left( {D_{i} } \right)\prime }} ,{\varvec{F}}_{{\text{e}}}^{{\left( {D_{j} } \right)\prime }} } \right)\overrightarrow {{\left( {n{ - }{1}} \right)}}^{\mathbf{T}} \hfill \\ d\left( {f_{{\text{e}}}^{{\left( { * ,m} \right)}} } \right) = {\varvec{S}}\left( {{\varvec{F}}_{{\text{e}}}^{{\left( {D_{i} } \right)}} ,{\varvec{F}}_{{\text{e}}}^{{\left( {D_{l} } \right)}} } \right)\overrightarrow {\left( n \right)}^{\mathbf{T}} - {\varvec{S}}\left( {{\varvec{F}}_{{\text{e}}}^{{\left( {D_{i} } \right)\prime }} ,{\varvec{F}}_{{\text{e}}}^{{\left( {D_{l} } \right)\prime }} } \right)\overrightarrow {{\left( {n{ - }{1}} \right)}}^{\mathbf{T}} \hfill \\ \end{gathered} \right.$$

If the extracted feature $f_{{\text{e}}}^{{\left( { * ,m} \right)}}$ satisfies the EFS criterion, then there is

14 $$\left\{ {\begin{array}{*{20}l} {\frac{1}{M}\sum\nolimits_{k} {\frac{{\sum\nolimits_{i} {\sum\nolimits_{j} {s\left( {f_{{\text{e}}}^{{\left( { * ,m} \right)}} } \right)} } }}{{\left| {B_{k} } \right|^{2} }}} > 0} \hfill \\ {\frac{1}{M}\sum\nolimits_{k} {\frac{{\sum\nolimits_{i} {\sum\nolimits_{l} {d\left( {f_{{\text{e}}}^{{\left( { * ,m} \right)}} } \right)} } }}{{\left| {B_{k} } \right|\left| {{\mathbf{B}} - \left\{ {B_{k} } \right\}} \right|}}} < 0} \hfill \\ \end{array} } \right. \Leftrightarrow \left\{ {\begin{array}{*{20}l} {\frac{1}{M}\sum\nolimits_{k} {\frac{{\sum\nolimits_{i} {\sum\nolimits_{j} {s\left( {f_{{\text{e}}}^{{\left( { * ,m} \right)}} } \right)} } }}{{\left| {B_{k} } \right|^{2} }}} > 0} \hfill \\ {\frac{1}{M}\sum\nolimits_{k} {\frac{{\sum\nolimits_{i} {\sum\nolimits_{l} {d\left( {f_{{\text{e}}}^{{\left( { * ,m} \right)}} } \right)} } }}{{\left| {B_{k} } \right|\left| {{\mathbf{B}} - \left\{ {B_{k} } \right\}} \right|}}} < 0} \hfill \\ {\frac{1}{M}\sum\nolimits_{k} {\frac{{\sum\nolimits_{i} {\sum\nolimits_{j} {s\left( {f_{{\text{e}}}^{{\left( { * ,m} \right)}} } \right)} } }}{{\left| {B_{k} } \right|^{2} }}} > \frac{\lambda }{M}\sum\nolimits_{k} {\frac{{\sum\nolimits_{i} {\sum\nolimits_{l} {d\left( {f_{{\text{e}}}^{{\left( { * ,m} \right)}} } \right)} } }}{{\left| {B_{k} } \right|\left| {{\mathbf{B}} - \left\{ {B_{k} } \right\}} \right|}}} } \hfill \\ \end{array} } \right.$$

or

15 $$\left\{ {\begin{array}{*{20}l} {\frac{1}{M}\sum\nolimits_{k} {\frac{{\sum\nolimits_{i} {\sum\nolimits_{j} {s\left( {f_{{\text{e}}}^{{\left( { * ,m} \right)}} } \right)} } }}{{\left| {B_{k} } \right|^{2} }}} > 0} \hfill \\ {\frac{1}{M}\sum\nolimits_{k} {\frac{{\sum\nolimits_{i} {\sum\nolimits_{l} {d\left( {f_{{\text{e}}}^{{\left( { * ,m} \right)}} } \right)} } }}{{\left| {B_{k} } \right|\left| {{\mathbf{B}} - \left\{ {B_{k} } \right\}} \right|}}} > 0} \hfill \\ {\frac{1}{M}\sum\nolimits_{k} {\frac{{\sum\nolimits_{i} {\sum\nolimits_{j} {s\left( {f_{{\text{e}}}^{{\left( { * ,m} \right)}} } \right)} } }}{{\left| {B_{k} } \right|^{2} }}} > \frac{\lambda }{M}\sum\nolimits_{k} {\frac{{\sum\nolimits_{i} {\sum\nolimits_{l} {d\left( {f_{{\text{e}}}^{{\left( { * ,m} \right)}} } \right)} } }}{{\left| {B_{k} } \right|\left| {{\mathbf{B}} - \left\{ {B_{k} } \right\}} \right|}}} } \hfill \\ \end{array} } \right.$$

In (14) and (15), $\lambda > 0$. Combining Eqs. (5), (6), (14) and (15), we obtain (16).

16 $$\left\{ {\begin{array}{*{20}l} {\frac{1}{M}\sum\nolimits_{k} {\frac{{\sum\nolimits_{i} {\sum\nolimits_{j} {s\left( {f_{{\text{e}}}^{{\left( { * ,m} \right)}} } \right)} } }}{{\left| {B_{k} } \right|^{2} }}} > 0} \hfill \\ {\frac{1}{M}\sum\nolimits_{k} {\frac{{\sum\nolimits_{i} {\sum\nolimits_{j} {s\left( {f_{{\text{e}}}^{{\left( { * ,m} \right)}} } \right)} } }}{{\left| {B_{k} } \right|^{2} }}} > \frac{\lambda }{M}\sum\nolimits_{k} {\frac{{\sum\nolimits_{i} {\sum\nolimits_{l} {d\left( {f_{{\text{e}}}^{{\left( { * ,m} \right)}} } \right)} } }}{{\left| {B_{k} } \right|\left| {{\mathbf{B}} - \left\{ {B_{k} } \right\}} \right|}}} } \hfill \\ \end{array} } \right. \Leftrightarrow \left\{ {\begin{array}{*{20}l} {\varphi \left( {f_{{\text{e}}}^{{\left( { * ,m} \right)}} } \right) > 0} \hfill \\ {\varphi \left( {f_{{\text{e}}}^{{\left( { * ,m} \right)}} } \right) > \lambda \delta \left( {f_{{\text{e}}}^{{\left( { * ,m} \right)}} } \right)} \hfill \\ \end{array} } \right.$$

Equation (16) shows that if $f_{{\text{e}}}^{{\left( { * ,m} \right)}}$ satisfies the EFS criterion, then (16) is correct. Similarly, if (16) is correct, $f_{{\text{e}}}^{{\left( { * ,m} \right)}}$ satisfies the EFS criterion.

In Recognizer, we use RQB rule to obtain the value of ${\chi }_{\mathrm{rqb}}$ of extracted feature. When the value of ${\chi }_{\mathrm{rqb}}$ is greater than 0, the extracted feature will be selected as brand feature. The larger the ${\chi }_{\mathrm{rqb}}$ value, the greater the feature weight. We can obtain (17) according to the RQB rule (8).

17 $$\chi_{{{\text{rqb}}}} \left( {f_{{\text{e}}}^{{\left( { * ,m} \right)}} } \right) > 0 \Leftrightarrow \left\{ {\begin{array}{*{20}l} {\varphi \left( {f_{{\text{e}}}^{{\left( { * ,m} \right)}} } \right) \ge 0} \hfill \\ {\varphi \left( {f_{{\text{e}}}^{{\left( { * ,m} \right)}} } \right) > \left( {1 - \alpha } \right)\alpha^{ - 1} \delta \left( {f_{{\text{e}}}^{{\left( { * ,m} \right)}} } \right)} \hfill \\ \end{array} } \right.$$

That means $\chi_{{{\text{rqb}}}} (f_{{\text{e}}}^{{\left( { * ,m} \right)}} ) > 0$ is equivalent to that $f_{{\text{e}}}^{{\left( { * ,m} \right)}}$ satisfies the EFS criterion.

The above analysis shows that RQB rule comply with EFS criteria, RFBR strategy can be used to evaluate the role of each feature in brand recognition, and it is reasonable to use RFBR strategy to select brand features. The value of features can be a significant reference for the selection of brand feature.

### Rationality analysis of RFMR strategy

Because one model only corresponds to one mobile device, there is no similarity between devices with same model. So, it is unreasonable to use RFBR strategy for model feature selection. For that, we use RFMR strategy to quantify the role of each feature in model recognition, and to select model features and determine weights. According to the EFS criterion, if one feature can be selected as model feature, the feature should be helpful to distinguish different models of device with same brand. This means that the similarity between two different model devices with same brand could be decreased after using this feature.

Assuming that $f_{{\text{e}}}^{{\left( { * ,1} \right)}} ,f_{{\text{e}}}^{{\left( { * ,2} \right)}} ,f_{{\text{e}}}^{{\left( { * ,3} \right)}} , \ldots ,f_{{\text{e}}}^{{\left( { * ,n} \right)}}$ are all extraction features, then extract the feature vector ${\mathbf{F}}_{{\text{e}}} = [f_{{\text{e}}}^{{\left( { * ,1} \right)}} ,f_{{\text{e}}}^{{\left( { * ,2} \right)}} ,f_{{\text{e}}}^{{\left( { * ,3} \right)}} , \ldots ,f_{{\text{e}}}^{{\left( { * ,n} \right)}} ]$. $\forall B_{k} \in {\mathbf{B}}$, $\forall D_{i} ,D_{j} \in B_{k}$, ${\varvec{F}}_{{\text{e}}}^{{\left( {D_{i} } \right)\prime }} = {\varvec{F}}_{{\text{e}}}^{{\left( {D_{i} } \right)}} \backslash f_{{\text{e}}}^{{\left( {D_{i} ,m} \right)}}$, ${\varvec{F}}_{{\text{e}}}^{{\left( {D_{j} } \right)\prime }} = {\varvec{F}}_{{\text{e}}}^{{\left( {D_{j} } \right)}} \backslash f_{{\text{e}}}^{{\left( {D_{j} ,m} \right)}}$. For each extracted feature $f_{{\text{e}}}^{{\left( { * ,m} \right)}}$ ($1 \le m \le n$), the similarity increments $s(f_{{\text{e}}}^{{\left( { * ,m} \right)}} )$ between any two devices with same brand is shown in (18).

18 $$s\left( {f_{{\text{e}}}^{{\left( { * ,m} \right)}} } \right) = {\varvec{S}}\left( {{\varvec{F}}_{{\text{e}}}^{{\left( {D_{i} } \right)}} ,{\varvec{F}}_{{\text{e}}}^{{\left( {D_{j} } \right)}} } \right)\overrightarrow {\left( n \right)}^{\mathbf{T}} - {\varvec{S}}\left( {{\varvec{F}}_{{\text{e}}}^{{\left( {D_{i} } \right)\prime }} ,{\varvec{F}}_{{\text{e}}}^{{\left( {D_{j} } \right)\prime }} } \right)\overrightarrow {{\left( {n - {1}} \right)}}^{\mathbf{T}}$$

If $f_{{\text{e}}}^{{\left( { * ,m} \right)}}$ satisfies the EFS criterion, then

19 $$M^{ - 1} \sum\nolimits_{k} {\left( {\left| {B_{k} } \right|^{{{ - }2}} \sum\nolimits_{i} {\sum\nolimits_{j} {s\left( {f_{{\text{e}}}^{{\left( { * ,m} \right)}} } \right)} } } \right)} < 0 \Leftrightarrow \varphi \left( {f_{{\text{e}}}^{{\left( { * ,m} \right)}} } \right) < 0$$

Equation (19) shows that if $f_{{\text{e}}}^{{\left( { * ,m} \right)}}$ satisfies the EFS criterion, then (19) is correct. Similarly, if (19) is correct, $f_{{\text{e}}}^{{\left( { * ,m} \right)}}$ satisfies the EFS criterion.

In Recognizer, we use RQM rule to obtain the value of ${\chi }_{\mathrm{rqm}}$ of extracted feature. When the value of ${\chi }_{\mathrm{rqm}}$ is greater than 0, the extracted feature will be selected as model feature. The larger the ${\chi }_{\mathrm{rqm}}$ value, the greater the feature weight. We can obtain (20) according to the RQM rule (10).

20 $$\chi_{{{\text{rqm}}}} \left( {f_{{\text{e}}}^{{\left( { * ,m} \right)}} } \right) > 0 \Leftrightarrow \varphi \left( {f_{{\text{e}}}^{{\left( { * ,m} \right)}} } \right) < 0$$

That means $\chi_{{{\text{rqm}}}} (f_{{\text{e}}}^{{\left( { * ,m} \right)}} ) > 0$ is equivalent to that $f_{{\text{e}}}^{{\left( { * ,m} \right)}}$ satisfies the EFS criterion.

At the same time, the incremental standard deviation of intra-brand similarity is another factor to assess the role of extracted feature in model recognition. According to (10), when the $\varphi$ values of two brand features are same, the bigger the $\gamma$ is, the smaller the ${\chi }_{\mathrm{rqm}}$ is. Since the standard deviation can be used to measure the degree of dispersion of data, the smaller the standard deviation, the more stable the data. Thus, when $\varphi (f_{{\text{e}}}^{{\left( { * ,i} \right)}} ) = \varphi (f_{{\text{e}}}^{{\left( { * ,j} \right)}} )$, if $\gamma (f_{{\text{e}}}^{{\left( { * ,i} \right)}} ) < \gamma (f_{{\text{e}}}^{{\left( { * ,j} \right)}} )$, $\chi_{{{\text{rqm}}}} (f_{{\text{e}}}^{{\left( { * ,i} \right)}} ) > \chi_{{{\text{rqm}}}} (f_{{\text{e}}}^{{\left( { * ,j} \right)}} )$. It shows that compared with $f_{{\text{e}}}^{{\left( { * ,j} \right)}}$, $f_{{\text{e}}}^{{\left( { * ,i} \right)}}$ can stably reduce the similarity between any two devices with same brand but different models. Thus, compared with $f_{{\text{e}}}^{{\left( { * ,j} \right)}}$, $f_{{\text{e}}}^{{\left( { * ,i} \right)}}$ plays a better role in model recognition.

The above analysis shows that RQM rule comply with EFS criteria, RFMR strategy can be used to evaluate the role of each feature in model recognition, and it is reasonable to use RFMR strategy to select model features. The value of features can be a significant reference for the selection of model feature.

## Results and analysis of experiments

In this section, we first introduce our experimental dataset. On this dataset, we carry out three experiments: (1) experiment on selecting brand features and determining weights, (2) experiment on selecting model features and determining weights, (3) experiment on device recognition using Recognizer and other methods.

### Experimental dataset

In this section, the experimental dataset includes 587 models of mobile phone devices from 17 brands. The brands and models of mobile devices are shown in Table 1.

**Table 1:** Brands and models of devices.

Brand	Model
Apple	iPhone 11, iPhone 11 Pro, iPhone 11 Pro Max, iPhone 12, iPhone 12 mini, iPhone 12 Pro, iPhone 12 Pro Max, iPhone SE 2, iPhone XR, iPhone XS, iPhone XS Max
HONOR	20, 30, 50, 10, 10 Lite, 10X Lite, 20 Lite, 20 Pro, 20i, 20S, 30 Pro, 30 Pro +, 30 Youth, 30i, 30S, 50 Pro, 50 SE, 8S, 8X, 8X Max, 9i, 9X, 9X Pro, Magic 2, Magic 2 3D, Magic3, Magic3 Pro, Magic3 Pro +, Note 10, Play, Play 20, Play 3, Play 3E, Play 4, Play 4 Pro, Play 4 T, Play 4 T Pro, Play 5 5G, Play 5 T, Play 5 T Pro, Play 5 T Youth, Play 7A, Play 7C, Play 8A, Play 8C, Play 9A, V20, V30, V30 Pro, V40, V40 Lite, X10, X10 Max, X20, X20 SE
Huawei	enjoy 10, enjoy 10 Plus, enjoy 10e, enjoy 10S, enjoy 20, enjoy 20 Plus, enjoy 20 Pro, enjoy 20 SE, enjoy 8, enjoy 9, enjoy 9 Plus, enjoy 9e, enjoy MAX, enjoy Z, Maimang 7, Maimang 8, Maimang 9, Mate 20, Mate 20 Pro, Mate 20 RS Porsche, Mate 20 X, Mate 30, Mate 30 Pro, Mate 30 RS Porsche, Mate 30E Pro 5G, Mate 40, Mate 40 Pro, Mate 40 Pro +, Mate 40 RS Porsche, Mate 40E, Mate RS Porsche, Mate X, Mate X2, Mate Xs, nova 3, nova 3e, nova 3i, nova 4, nova 4e, nova 5, nova 5 Pro, nova 5i, nova 5i Pro, nova 5Z, nova 6, nova 6 5G, nova 6 SE, nova 7, nova 7 Pro, nova 7 SE, nova 8, nova 8 5G, nova 8 Pro, nova 8 SE, nova 8 SE Youth, nova 8i, nova 9, nova 9 Pro, P20, P20 Lite, P20 Pro, P30, P30 Pro, P40, P40 Pro, P40 Pro + , P50, P50 Pro, Y7p, Y8s, Y9a
iQOO	3, 5, 7, 8, 1, 5 Pro, 8 Pro, Neo, Neo 3, Neo 5, Neo 5 Lite, Neo 855, Neo 855 Racing, Pro, U1, U1, U1x, U3, U3x, U3x Standard, Z1, Z1x, Z3, Z5
Lenovo	K5, K5 Note, K5 Play, K5 Pro, Legion 2 Pro, Legion Pro, Lemon K12, Lemon K12 Pro, S5, S5 Pro, S5 Pro GT, Z5, Z5 Pro, Z5s, Z6, Z6 Pro, Z6 Youth
Meizu	15, 17, 18, 15 Plus, 16, 16 Plus, 16 s, 16 s Pro, 16 T, 16x, 16Xs, 17 Pro, 18 Pro, 18 s, 18 s Pro, 18x, E3, M15 Lite, M6s, M6T, Note 8, Note 9, V8, V8 Pro, X8
Motorola	E5 Plus, Edge Light, Edge S, Edge S Pro, Edge + , G 5G Plus, G50, G50 5G, G7 Plus, Z Play, Z3, One Hyper, One Zoom, P30, P30 Note, P30 Play, P50
Nokia	3.4, 8.3, 6.2, 7 Plus, 8 Sirocco, 9 PureView, C10, C2, C20, C3, G10, G20, X10, X20, X5, X6 2018, X7 2018, X71
Nubia	N3, Play, Red Magic, Red Magic 3, Red Magic 5G, Red Magic 5S, Red Magic 6, Red Magic 6 Pro, Red Magic 6R, Red Magic Mars, V18, X, Z18, Z18 mini, Z20, Z30 Pro
Oneplus	6, 7, 8, 9, 6T, 6T Mclaren, 7 Pro, 7T, 7T Pro, 7T Pro Mclaren, 8 Pro, 8T, 9 Pro, 9R, Nord, Nord 2 5G, Nord CE 5G, Nord N10 5G
OPPO	A1, A11, A11k, A15, A15s, A3, A32, A35, A5, A52, A55, A7, A72, A72 5G, A74, A74 5G, A7x, A8, A91, A92s, A93, A93 5G, A93s, A94, A94 5G, A95, Ace, Ace2, F19 Pro, Find X, Find X Lamborghini, Find X2, Find X2 Lite, Find X2 Pro, Find X3, Find X3 Lite, Find X3 Neo, Find X3 Pro, K1, K3, K5, K7, K7x, K9, K9 Pro, R15, R15x, R17, R17 Pro, Reno, Reno 2, Reno 2 Z, Reno 3, Reno 3 Pro, Reno 4, Reno 4 Pro, Reno 4 SE, Reno 5, Reno 5 K, Reno 5 Lite, Reno 5 Pro, Reno 5 Pro + , Reno 5 Z, Reno 6, Reno 6 Pro, Reno 6 Pro + , Reno Ace, Reno Z
Realme	6, 7, 8, 3 Pro, 6 Pro, 6i, 6 s, 7 Pro, 7i, 8 Pro, C15, C20, C21, C25, C3, GT, GT Explorer Master, GT Master, GT Neo, GT Neo Flash, Narzo 10, Narzo 30, Narzo 30 Pro, Narzo 30A, Q, Q2, Q2 Pro, Q2i, Q3, Q3 Pro, Q3 Pro Carnival, Q3i, V13, V15, V3, V5, X, X Youth, X2, X2 Pro, X3, X3 Pro, X50, X50 Pro, X50 Pro Player, X50m, X7, X7 Pro, X7 Pro Ultra
Redmi	6, 7, 8, 9, 10X, 10X Pro, 6A, 7A, 8A, 8A Pro, 9A, K20, K20 Pro, K30, K30 Pro, K30i, K30S, K40, K40 Gaming, K40 Pro, K40 Pro + , Note 10 4G, Note 10 5G, Note 10 Pro, Note 10 Pro Max, Note 10 s, Note 5, Note 6 Pro, Note 7, Note 7 Pro, Note 7S, Note 8, Note 8 Pro, Note 9 4G, Note 9 5G, Note 9 Pro, Note 9 Pro Max, Note 9S, Note 9 T, S2
Samsung	Galaxy A02, Galaxy A02S, Galaxy A12, Galaxy A20s, Galaxy A32, Galaxy A50s, Galaxy A51, Galaxy A52, Galaxy A6s, Galaxy A70, Galaxy A70s, Galaxy A71, Galaxy A80, Galaxy A8s, Galaxy A90, Galaxy A9s, Galaxy F12, Galaxy F52, Galaxy F62, Galaxy Fold, Galaxy M12, Galaxy M30s, Galaxy M31s, Galaxy Note 10, Galaxy Note 10 +, Galaxy Note 20, Galaxy Note 20 Ultra, Galaxy Note 9, Galaxy S10, Galaxy S10 +, Galaxy S10e, Galaxy S20, Galaxy S20 FE, Galaxy S20 Ultra, Galaxy S20 +, Galaxy S21, Galaxy S21 FE, Galaxy S21 Ultra, Galaxy S21 +, Galaxy S9, Galaxy S9 +, Galaxy XCover 5, Galaxy Z Flip, Galaxy Z Flip3, Galaxy Z Fold2, Galaxy Z Fold3
VIVO	NEX 3, NEX 3S, NEX Dual Display, S1, S1 Pro, S10, S10 Pro, S5, S6, S7, S7e, S9, S9e, U3, U3x, V19, X20 Plus UD, X21, X21i, X23, X27, X27 Pro, X30, X30 Pro, X50, X50 Pro, X50 Pro +, X60, X60 Pro, X60 Pro +, X60t, Y12s, Y20G, Y20i, Y30, Y30g, Y31s, Y3s, Y50, Y51s, Y52s, Y52s t1, Y53s, Y69, Y70s, Y70t, Y71, Y73s, Y83, Y91, Y93, Y93s, Y97, Z1, Z1 Lite, Z1i, Z3, Z3i, Z5, Z5i, Z5x, Z6
Xiaomi	8, 9, 10, 11, 10 Lite, 10 Pro, 10S, 11 Lite, 11 Pro, 11 Ultra, 11i, 11X, 11X Pro, 6X, 8 Lite, 8 SE, 9 Pro, 9 SE, A2, A3, CC9, CC9 Pro, CC9e, Max 3, Mi Play, MIX 2s, MIX 3, MIX 4, MIX FOLD, POCO F3, POCO M3, POCO X3 Pro
ZTE	AXON 10 Pro, AXON 11, AXON 11 SE, AXON 20, AXON 30, AXON 30 Pro, AXON 30 Ultra, AXON 9 Pro, Axon M, Blade A7S, Blade 20, Blade A7, Blade V10, Blade V2020, S30, S30 Pro, S30 SE, V9

We extract 21 common attributes from devices in Table 1 as extracted features: device dimensions, device weight, display size, screen-to-body ratio, resolution, display density, chipset, GPU, internal memory, memory card, operating system, battery capacity, battery charging, selfie camera, main camera, network technology, SIM, WLAN, NFC, Bluetooth, GPS. According to the form of extracted features, these features are numerical features: device dimensions, device weight, display size, screen-to-body ratio, resolution, display density, battery capacity, NFC, Bluetooth (where the device dimensions and resolution are multi-dimensional numerical features). And those extracted features are string features: chipset, GPU, internal memory, memory card, operating system, battery charging, selfie camera, main camera, network technology, SIM, WLAN, GPS (among which, battery charging, selfie camera, main camera, WLAN and GPS are multi-strings features). In our experiments, we set *ε* = 1 in calculating similarity between two numerical features.

Our dataset contains two parts: knowledge set and target set. The knowledge set and target set all include 587 different models of mobile devices, and the difference is: for any feature of mobile phone device, if there are multiple possible feature values, the target set only includes one possible value of the device. This means that the size of knowledge set is 587, but the size of target set is not less than 587. For example, the internal memory of iPhone 12 Pro has three possible values: “6 GB + 128 GB”, “6 GB + 256 GB” and “6 GB + 512 GB”.

Thus, in the knowledge set, the internal memory value of iPhone 12 Pro is “6 GB + 128 GB; 6 GB + 256 GB; 6 GB + 512 GB”. But in the target set, there will be three items about “iPhone 12 Pro” devices at least, and the internal memory of those “iPhone 12 Pro” is one of “6 GB + 128 GB”, “6 GB + 256 GB” and “6 GB + 512 GB”. The device item number of different brands in knowledge set and target set are shown in Table 2.

**Table 2:** Device number of different brands in knowledge set and target set.

Brand	Knowledge set	Target set	Brand	Knowledge set	Target set
Apple	11	33	Oneplus	18	41
HONOR	55	135	OPPO	68	152
Huawei	71	188	Realme	49	130
iQOO	24	78	Redmi	40	1498
Lenovo	17	57	Samsung	46	101
Meizu	25	65	vivo	62	161
Motorola	17	30	Xiaomi	32	136
NOKIA	18	38	ZTE	18	43
Nubia	16	45	Total	587	2931

### Selecting brand features and determining weights

**Figure 2:**
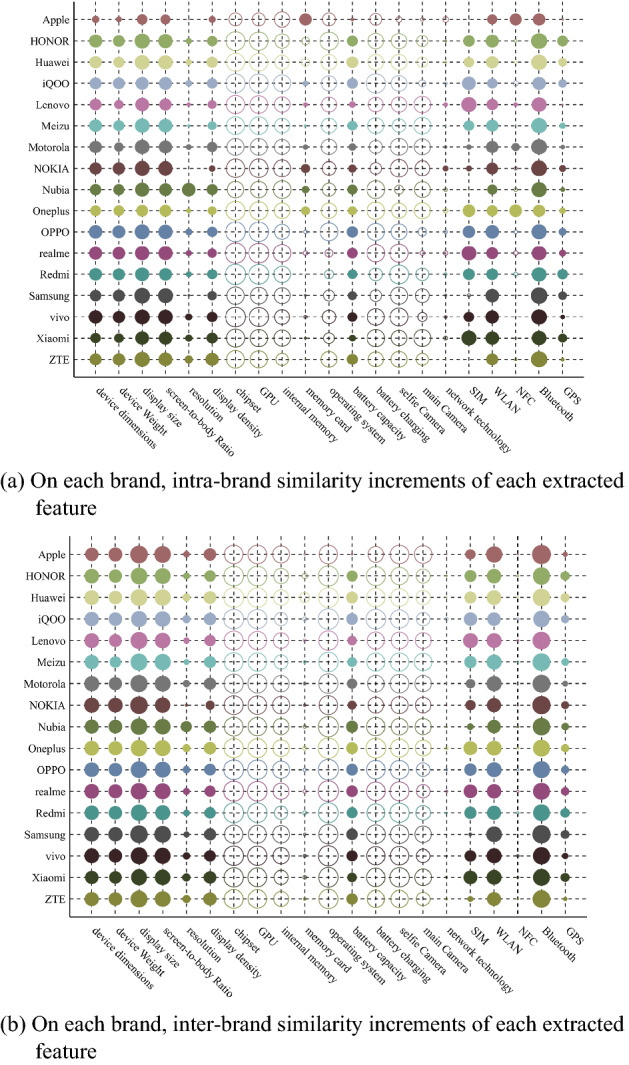
The effect of each extracted feature on the similarity of the devices with same brand and different brands. The size of circle presents absolute value of similarity increment. The bigger the circle is, the larger the value is. The solid circle indicates a positive value, and the hollow circle indicates a negative value.

For each extracted feature, we calculate the average intra-brand similarity increment between two devices with same brand, and the inter-brand similarity increment between two devices with different brands. The effect of each extracted feature on the similarity of the devices with same brand and different brands is shown in Fig. 2.

As can be seen from Fig. 2, some extracted features, such as device dimensions, device weight, screen-to-body ratio, etc., can not only increase intra-brand similarity, but also increase inter-brand similarity. The reason may be that when manufacturers design mobile phones, they usually draw on the attributes of other brands, resulting in the similarity in some extracted features of phones with different brands. Therefore, in the acceptable case of EFS criterion, the extracted features, such as device dimensions, device weight and screen-to-body ratio, may be able to be selected as brand features.

We set $\alpha =0.8$. According to (5), (6) and (8), we calculate the average intra-brand similarity increment ($\varphi$), the average inter-brand similarity increment ($\delta$), value of ${\chi }_{\mathrm{rqb}}$, and normalized weight (${\omega }_{\mathrm{b}}$). The results are shown in Table 3.

**Table 3:** The values of $\varphi$, $\delta$, ${\chi }_{\mathrm{rqb}}$, and ${\omega }_{\mathrm{b}}$ of each extracted feature.

Extraction Feature	$\varphi$(× 10^–3^)	$\delta$(× 10^–3^)	${\chi }_{\mathrm{rqb}}$(× 10^–4^)	${\omega }_{\mathrm{b}}$(%)
Device dimensions	15.368	18.155	86.634	9.948
Device weight	14.076	16.796	79.015	9.073
Display size	18.227	21.312	103.194	11.849
Screen-to-body ratio	17.354	20.055	98.720	11.335
Resolution	6.641	8.544	36.042	4.139
Display density	12.320	14.761	69.034	7.927
Chipset	− 24.977	− 25.671	0	0
GPU	− 24.384	− 25.219	0	0
Internal memory	− 21.365	− 20.942	0	0
Memory card	2.084	− 4.876	26.426	3.034
Operating system	− 18.941	− 26.521	0	0
Battery capacity	11.486	12.833	66.217	7.603
Battery charging	− 21.049	− 24.086	0	0
Selfie camera	− 20.891	− 24.156	0	0
Main camera	− 16.594	− 20.205	0	0
Network technology	− 2.779	− 1.674	0	0
SIM	11.063	13.638	61.225	7.030
WLAN	15.449	18.349	86.890	9.977
NFC	0.159	− 1.902	5.076	0.583
Bluetooth	19.028	22.197	107.827	12.381
GPS	7.727	8.609	44.595	5.121

Table 3 shows that those extracted features are selected as brand features: device dimensions, device weight, display size, screen-to-body ratio, resolution, display density, memory card, battery capacity, SIM, WLAN, NFC, Bluetooth, and GPS. In device recognition experiment in this paper, those brand features will be used to recognize the brand of target device.

### Selecting model features and determining weights

**Figure 3:**
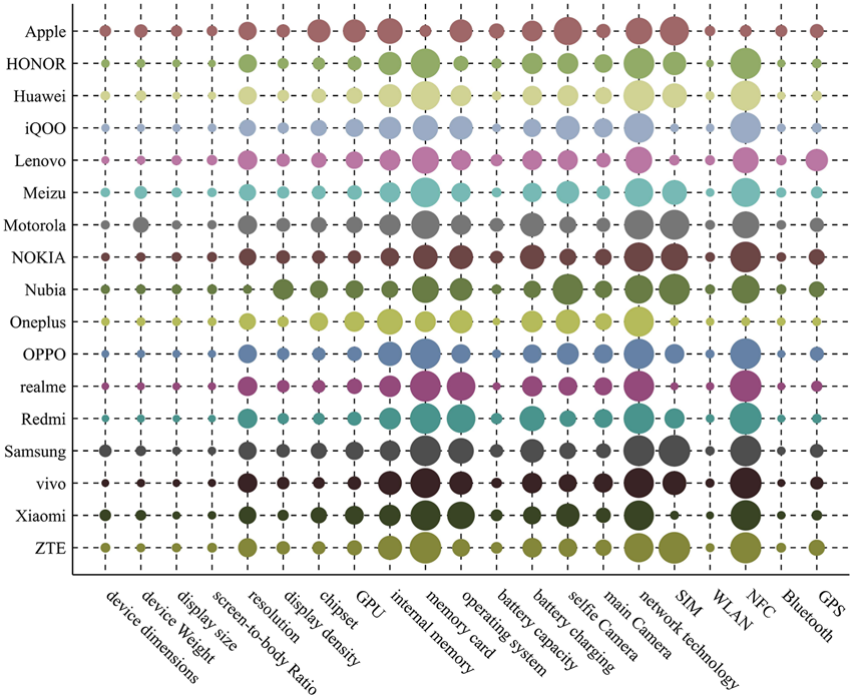
On each brand, the standard deviation of intra-brand similarity increments of different extracted feature. The size of circle presents absolute value of similarity increment. The bigger the circle is, the larger the value is.

For each extracted feature, we calculate the standard deviation of intra-brand similarity increment, and the result is shown in Fig. 3.

For two extracted features, when average intra-brand similarity increments ($\varphi$) are same, the extracted feature with smaller standard deviation could stably decrease intra-brand similarity for all brand.

We set $\beta =0.8$. For each extracted feature, according to (9) and (10), we calculate the standard deviation of intra-brand similarity increment ($\gamma$), value of ${\chi }_{\mathrm{rqm}}$, and normalized weight (${\omega }_{\mathrm{m}}$). The calculation results are shown in Table 4. The $\varphi$ value of each extracted feature has been calculated in subsection Selecting brand features and determining weights, thus, we directly use the $\varphi$ value in subsection Selecting brand features and determining weights when calculating ${\chi }_{\mathrm{rqm}}$ here.

**Table 4:** The values of $\gamma$, ${\chi }_{\mathrm{rqm}}$, and ${\omega }_{\mathrm{m}}$ of each extracted feature.

Extraction feature	$\gamma$(× 10^–3^)	${\chi }_{\mathrm{rqm}}$(× 10^–4^)	${\omega }_{\mathrm{m}}$(%)
Device dimensions	6.189	0	0
Device weight	6.855	0	0
Display size	5.936	0	0
Screen-to-body ratio	5.979	0	0
Resolution	12.862	0	0
Display density	9.034	0	0
Chipset	10.780	178.253	18.019
GPU	11.921	171.229	17.309
Internal memory	16.212	138.496	14.000
Memory card	20.397	0	0
Operating system	16.566	118.396	11.968
Battery capacity	7.536	0	0
Battery charging	15.169	138.055	13.956
Selfie camera	15.493	136.145	13.763
Main camera	12.040	108.668	10.985
Network technology	21.920	− 21.607	0
SIM	15.560	0	0
WLAN	6.109	0	0
NFC	20.628	0	0
Bluetooth	6.114	0	0
GPS	9.172	0	0

Table 4 shows that those extracted features are selected as model features: chipset, GPU, internal memory, operating system, battery charging, selfie camera, main camera. In device recognition experiment in this paper, those model features will be used to recognize the model of target device.

According to subsections Selecting brand features and determining weights and Selecting model features and determining weights, we build a feature set including 20 features. Those features are: device dimensions, device weight, display size, screen-to-body ratio, resolution, display density, chipset, GPU, internal memory, memory card, operating system, battery capacity, battery charging, selfie camera, main camera, SIM, WLAN, NFC, Bluetooth, GPS.

### Device recognition using Recognizer and other methods

In this subsection, Recognizer, ProfilIoT^13^, MSA^20^ and ByteIoT^21^ are used to recognize the brand and model of devices in the target set, respectively.

Firstly, all 20 features in feature set are used in model recognition of mobile device. The recognition accuracy values of four methods are shown in Table 5.

**Table 5:** Recognition accuracy values of four methods in different brands.

Brand	Target set	Recognizer		ProfilIoT^13^		MSA^20^		ByteIoT^21^	
		Brand Acc	Model Acc	Brand Acc	Model Acc	Brand Acc	Model Acc	Brand Acc	Model Acc
Apple	33	100% (33)	72.73% (24)	100% (33)	63.64% (21)	100% (33)	63.64% (21)	100% (33)	63.64% (21)
HONOR	135	100% (135)	97.78% (132)	100% (135)	84.44% (114)	100% (135)	87.41% (118)	100% (135)	84.44% (114)
Huawei	188	100% (188)	97.34% (183)	87.77% (165)	74.47% (140)	92.55% (174)	85.11% (160)	91.49% (172)	81.91% (154)
iQOO	78	100% (78)	100% (78)	100% (78)	89.74% (70)	100% (78)	94.87% (74)	100% (78)	94.87% (74)
Lenovo	57	100% (57)	100% (57)	100% (57)	100% (57)	100% (57)	100% (57)	100% (57)	100% (57)
Meizu	65	100% (65)	96.92% (63)	100% (65)	78.46% (51)	100% (65)	96.92% (63)	100% (65)	96.92% (63)
Motorola	30	100% (30)	100% (30)	100% (30)	90.00% (27)	100% (30)	96.67% (29)	100% (30)	90.00% (27)
NOKIA	38	100% (38)	100% (38)	100% (38)	89.47% (34)	100% (38)	89.47% (34)	100% (38)	89.47% (34)
Nubia	45	100% (45)	100% (45)	100% (45)	75.56% (34)	100% (45)	91.11% (41)	100% (45)	91.11% (41)
Oneplus	41	100% (41)	100% (41)	100% (41)	92.68% (38)	100% (41)	92.68% (38)	100% (41)	92.68% (38)
OPPO	152	100% (152)	100% (152)	100% (152)	79.61% (121)	100% (152)	94.08% (143)	100% (152)	93.42% (142)
realme	130	100% (130)	96.92% (126)	83.85% (109)	56.92% (74)	94.62% (123)	68.46% (89)	94.62% (123)	68.46% (89)
Redmi	1498	100% (1498)	100% (1498)	100% (1498)	87.38% (1309)	100% (1498)	92.72% (1389)	100% (1498)	92.72% (1389)
Samsung	101	100% (101)	100% (101)	100% (101)	82.18% (83)	100% (101)	99.01% (100)	100% (101)	90.10% (91)
vivo	161	99.38% (160)	99.38% (160)	94.41% (152)	72.67% (117)	94.41% (152)	80.12% (129)	94.41% (152)	79.50% (128)
Xiaomi	136	100% (136)	98.53% (134)	86.76% (118)	76.47% (104)	89.71% (122)	80.88% (110)	89.71% (122)	80.88% (110)
ZTE	43	100% (43)	97.67% (42)	95.35% (41)	83.72% (36)	100% (43)	88.37% (38)	100% (43)	88.37% (38)
Total	2931	99.97% (2930)	99.08% (2904)	97.51% (2858)	82.91% (2430)	98.50% (2887)	89.83% (2633)	98.43% (2885)	89.05% (2610)

In Table 5, “Brand Acc” is the brand recognition accuracy, and the value in parentheses below the accuracy value is the number of devices whose brand recognition results are correct in target set. “Model Acc” is the model recognition accuracy, and the value in parentheses below the accuracy value is the number of devices whose model recognition results are correct in target set. It is worth noting the model recognition result of device must be wrong, when brand recognition result is wrong.

Table 5 shows that: (1) Recognizer, ProfilIoT, MSA and ByteIoT can recognize the brand and model of target device using our features. (2) the model recognition accuracy of Apple’s mobile phone is significantly lower than other brands using Recognizer. So are the other three methods. We analyze model recognition results and find that Recognizer mistakenly recognized the phone model as other phone models in same series, such as recognizing “iPhone 11 Pro Max” as “iPhone 11 Pro”, “iPhone 12 mini” as “iPhone 12”, “iPhone XS Max” as “iPhone XS”. We check feature values and found that values of all model features between misrecognized device model and true device model are same. That may be the internal reason of model misrecognition. (3) For all mobile phones in 17 brands, the brand recognition accuracy and model recognition accuracy are 99.97% and 99.08%, higher than existing methods, respectively.

Compared with the physical attributes, traffic features of device can be obtained easier. In our feature set, the resolution, operation system, and GPU of device can be obtained in the normal traffic. When only using the three traffic features, for different brands, the model recognition accuracy of Recognizer is shown in Table 6.

**Table 6:** Model recognition accuracy of Recognizer using traffic features only.

Brand	Model Acc (%)	Brand	Model Acc (%)	Brand	Model Acc (%)
Apple	33.65	Motorola	35.84	Redmi	23.92
HONOR	41.85	NOKIA	37.53	Samsung	28.01
Huawei	23.65	Nubia	32.00	vivo	21.93
iQOO	35.89	Oneplus	29.59	Xiaomi	25.42
Lenovo	40.42	OPPO	21.51	ZTE	31.24
Meizu	34.17	Realme	17.04		

In Table 6, “Model Acc” is the model recognition accuracy. The results show that, when only using three traffic features, the model recognition accuracy of Recognizer is low. But we believe that more traffic features can improve the model recognition accuracy of Recognizer. Moreover, in some actual scenario, we can obtain some physical attributes of device (namely, all features in feature set may not be acquired simultaneously). Next, we use a part of features in the feature set to identify the brand and model of device.

**Figure 4:**
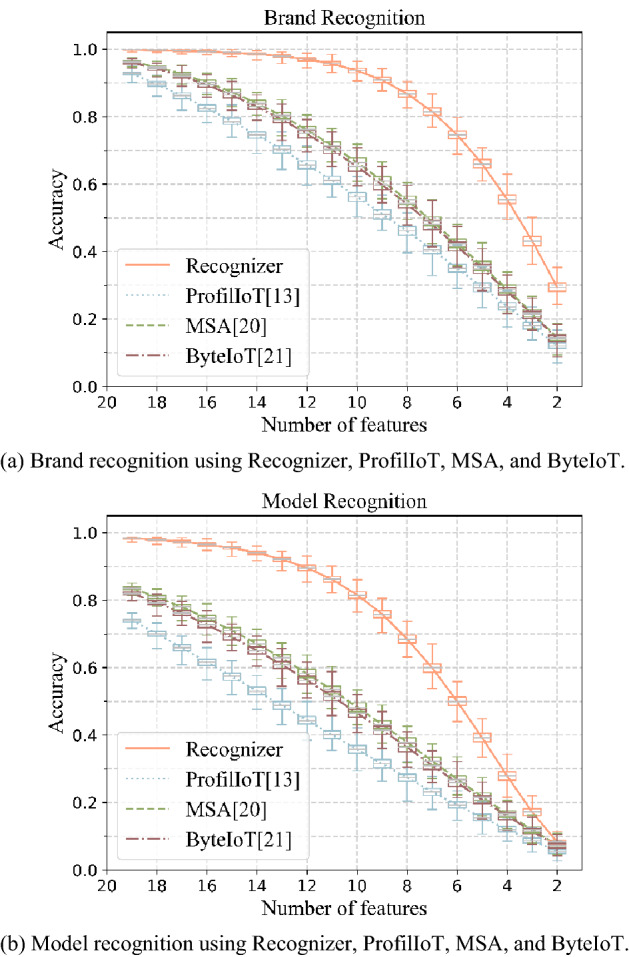
The relationship between recognition accuracy of the four methods and number of features. The box plot shows the maximum, minimum, Q1, Q3, and average of the recognition accuracy of Recognizer, ProfilIoT, MSA, and ByteIoT in 1000 sample sets in each number of features.

We gradually reduce the number of used features from 19 to 2 (1 reduction each time). For each specific number of features (*x*_0_), we randomly select × 0 features from 20 features of all devices in target set, and the other (20-*x*_0_) features of all target devices are set null. In this way, we build 1 sample set (the size of sample set is equal to target set). For each *x*_0_, we build 1000 sample sets. Finally, Recognizer, ProfilIoT, MSA, and ByteIoT are used to recognize the brand and model of device in sample set. The relationship between recognition accuracy of the four methods and number of features is shown in Fig. 4.

Figure 4 shows that: (1) brand recognition accuracy and model recognition accuracy of Recognizer, ProfilIoT, MSA, and ByteIoT both decrease as the number of features decreases, and the recognition accuracy of Recognizer is greater than ProfilIoT, MSA, or ByteIoT. (2) When the number of features is less than 9, as the number of features decreases, both the brand recognition accuracy and model recognition accuracy of Recognizer decrease rapidly. (3) When using 13 features, the average model recognition accuracy of Recognizer is 92.08%, an improvement of 29.26% over existing methods.

The above experimental results show that the model recognition accuracy of Recognizer is 99.08% (+ 9.25%↑) when using all 20 features in feature set. And when using any 13 features in feature set, the accuracy of Recognizer is 92.08% (+ 29.26%↑). The model recognition accuracy of Recognizer is higher than existing methods. This characteristic, that using a part of features in feature set also has a high recognition accuracy, is conducive to the widespread use of Recognizer.

## Conclusion

In this paper, we propose Recognizer, a method to recognize the model of mobile device based on weighted feature similarity. We build a feature set including 20 features firstly. Then, we design RFBR and RFMR strategies to select features from feature set, and determine the weight of each feature. Finally, for target mobile device, based on all or part features in feature set, Recognizer identifies the model of target mobile device. The experimental results show that not only Recognizer, but also existing methods can use the features in feature set to recognize the model of mobile device. And the model recognition accuracy of Recognizer is greater than other four methods. when using all features in feature set, the accuracy of Recognizer is 99.08% (+ 9.25%↑). And when using any 13 features in feature set, the accuracy of Recognizer is 92.08% (+ 29.26%↑). In the process of recognition, some physical attributes are used in Recognizer, and a few of these physical attributes may be obtained by in-touch. Therefore, compared with existing traffic-based methods, the range of applications of Recognizer is limited. In future work, how to use fewer and easier-to-obtain features to recognize the model of device will be an important research direction.

## Data Availability

The information of mobile device is obtained on https://www.gsmarena.com/search.php3.
